# Expression Concordance of 325 Novel RNA Biomarkers between Data Generated by NanoString nCounter and Affymetrix GeneChip

**DOI:** 10.1155/2019/1940347

**Published:** 2019-05-14

**Authors:** Lucas Delmonico, Said Attiya, Joan W. Chen, John C. Obenauer, Edward C. Goodwin, Marcia V. Fournier

**Affiliations:** ^1^Bioarray Genetics Inc., Farmington, 06032 Connecticut, USA; ^2^Rancho BioSciences, San Diego, 92127 California, USA

## Abstract

**Background:**

With the development of new drug combinations and targeted treatments for multiple types of cancer, the ability to stratify categories of patient populations and to develop companion diagnostics has become increasingly important. A panel of 325 RNA biomarkers was selected based on cancer-related biological processes of healthy cells and gene expression changes over time during nonmalignant epithelial cell organization. This “cancer in reverse” approach resulted in a panel of biomarkers relevant for at least 7 cancer types, providing gene expression profiles representing key cellular signaling pathways beyond mutations in “driver genes.” *Objective*. To further investigate this biomarker panel, the objective of the current study is to (1) validate the assay reproducibility for the 325 RNA biomarkers and (2) compare gene expression profiles side by side using two technology platforms.

**Methods and Results:**

We have mapped the 325 RNA transcripts and in a custom NanoString nCounter expression panel to be compared to all potential probe sets in the Affymetrix Human Genome U133 Plus 2.0. The experiments were conducted with 10 unique biological formalin-fixed paraffin-embedded (FFPE) breast tumor samples. Each site extracted RNA from four sections of 10-micron thick FFPE tissue over three different days by two different operators using an optimized standard operating procedure and quality control criteria. Samples were analyzed using mas5 in BioConductor and NanoStringNorm in R. Pearson correlation showed reproducibility between sites for all 60 samples with *r* = 0.995 for Affymetrix and *r* = 0.999 for NanoString. Correlation in multiple days and multiple users was for Affymetrix *r* = (0.962 − 0.999) and for NanoString *r* = (0.982 − 0.991).

**Conclusion:**

The 325 RNA biomarkers showed reproducibility in two technology platforms with moderate to high concordance. Future directions include performing clinical validation studies and generating rationale for patient selection in clinical trials using the technically validated assay.

## 1. Introduction

We developed a new panel of 325 RNA biomarkers selected based on cancer-related biological processes of healthy cells and gene expression changes over time during nonmalignant epithelial cell organization [1–5]. This approach based on gene expression patterns correlated to phenotypes of nonmalignant breast epithelial cells resulted in a panel of biomarkers with gene expression profiles in at least 7 cancer types and little overlap with 9 other widely used commercial gene panels analyzed to date (e.g., overlap with FoundationOne was 2% (lowest) and with Oncotype DX was 14% (highest)) (manuscript submitted). Moreover, the panel provided a new set of oncology biomarker-based gene expression profiles associated with clinical outcomes in multiple independent microarray datasets [1, 2].

In this context, preanalytic variables may interfere with the gene expression profiling results, for example, RNA integrity coming from formalin-fixed paraffin-embedded (FFPE) samples. In the present study, we validate the analytical performance to profile these 325 RNA biomarkers using a custom-designed NanoString assay in comparison to Affymetrix microarray gene chip.

FFPE breast tumor biopsies are routinely used in clinical practice, since the collection of fresh or frozen samples is not feasible for the set of histopathological tests to be performed. The quality of the total RNA extracted from FFPE samples is crucial for the evaluation of the gene expression profiles and depends on a series of factors such as the quality of the materials used for fixing, the quality of the material to be fixed, cross-liking, and degradation of paraffin-embedded genetic material over the years, among others [6]. In addition, different gene expression analysis technologies can generate divergent results across platforms. For example, Affymetrix GeneChip microarray used the photolithography technology that couples with fluorescence signal detection to measure the expression of genes that are amplified during sample preparation [7, 8]; on the other hand, the NanoString platform utilizes single-molecule imaging and counting without any amplification steps [9]. Further, among five platforms examined (Affymetrix GeneChip, NanoString nCounter™, Illumina whole genome RNA-Seq, Illumina targeted RNA-Seq, and Illumina stranded Total RNA-rRNA-depletion), NanoString provided a more faithful translation of a gene expression signature from FF (fresh frozen) to FFPE samples [10].

Based on this context, we aimed to compare the expression profiles of the 325 RNA biomarkers on the NanoString custom-designed assay against Affymetrix GeneChip. RNA extracted from 10 unique FFPE breast tumor biopsies by two different laboratories on three different days was analyzed with focus on the reproducibility of the assay in a total of 60 samples. The study showed that the custom-designed NanoString assay for the 325 RNA biomarkers is reproducible and showed moderate to high concordance to Affymetrix gene expression profiles.

## 2. Materials and Methods

### 2.1. Biological Samples

Ten unique FFPE breast tumor biopsies were analyzed, including five triple-negative (TNB) and five estrogen receptor-positive (ER+) breast cancer samples (Supplementary 1). The number of samples was chosen based on requirements by the College of American Pathologists (CAP) and Clinical Laboratory Improvement Amendments (CLIA). In total, 60 specimens were evaluated including RNA of each sample extracted by Bioarray (Laboratory 1) and LabCorp/Covance (Laboratory 2) over three different days, using the RNeasy FFPE kit (QIAGEN, Hilden, Germany), according to the manufacturer's protocol with modifications. For each RNA extraction, a histopathological evaluation was performed by an internal certified pathologist to confirm the tumor content. All 60 biological samples had quantification and quality (QC) results through the NanoDrop 1000 (Thermo Scientific, Wilmington, DE), RiboGreen (Life Technologies, Carlsbad, CA, USA), and Agilent Bioanalyzer (BA) (2100 Bioanalyzer Instrument, Santa Clara, CA, USA) (Supplementary 2).

### 2.2. Universal Human Reference RNA

The Universal Human Reference RNAs (UHR) (Agilent Technologies, TX, USA) were used as an amplification and hybridization positive control for the Affymetrix and NanoString assays.

### 2.3. NanoString Protocol: Sample Preparation and Hybridization

We designed a custom nCounter® assay (NanoString Technologies, Seattle, WA) for quantitative assessment of expression of 325 gene elements. Due to the change of analysis platforms, we designed the probes to hit the maximum number of validated transcripts and with the closest coverage of the probes analyzed in the Affymetrix platform. In addition to the 325 genes, there were 7 housekeeping genes, 6 positive control genes, and 8 negative control genes included in the same panel totaling in 346 genes analyzed. The full list with the 346 genes can be found in Supplementary 3. The appropriate mass of sample was prepared in a 96-well plate according to the specifications of the NanoString protocol. 5 *μ*L of each sample was mixed with 8 *μ*L of the hybridization cocktail (4 *μ*L of the reporter codeset and 4 *μ*L of hybridization buffer). 2 *μ*L of the capture codeset was added; the solution was mixed and spin down. It was placed in a 65°C thermocycler (Veriti Thermal Cycler, Applied Biosystems, Foster City, CA, USA) for 20 hours.

### 2.4. Preparation Station and Digital Analyzer

The samples were transferred to the preparation station with prepared reagent plates and a cartridge. The samples ran with the standard sensitivity for maximum binding to the cartridge. The preparation station ran approximately 3 hours.

The cartridges were transferred to the Digital Analyzer (NanoString Technologies, Seattle, WA) for analysis. A field of view (FOV) of 280 was used for the cartridges of the project, due to expected lower expression levels of the genes of interest. The Digital Analyzer ran approximately 2.5 hours for each cartridge.

### 2.5. nCounter™ Performance QC Metrics and Analysis

All of the cartridges used for the experiment were evaluated with the standard NanoString nCounter™ performance QC metrics (Supplementary 4). Any samples or cartridges that failed these metrics were repeated or flagged and removed from analysis. Raw data (RCC files) were received from NanoString and used directly as input for the open source R package, NanoStringNorm [11] for background correction and between-sample normalization.

### 2.6. Affymetrix NuGEN-FFPE Amplification and Affymetrix Hybridization

The biological samples were prepared such that 100 ng of mass was added to a 96-well plate and the standard NuGEN-FFPE WTA v3 (NuGEN Technologies Inc., San Carlos, CA, USA) amplification protocol was used. The samples were cleaned, following single primer isothermal amplification (SPIA), with 35 *μ*L of deionized water. The concentration and integrity of the samples were evaluated and then 5 *μ*g of mass was run through the standard NuGEN Biotin fragmentation and labeling protocol (NuGEN Technologies Inc., San Carlos, CA, USA).

Hybridization solution was added to the samples and 200 *μ*L of each sample was added to the Affymetrix cartridges (Affymetrix GeneChip Human Genome U133 Plus 2.0 array, GeneChip Expression Arrays, Santa Clara, CA) and placed in the hybridization oven at 65°C with 60 rotations per minute. The Affymetrix cartridges were hybridized for 18 hours, washed and stained in the Affymetrix Wash/Stain equipment, and then scanned.

### 2.7. Affymetrix Performance QC Metrics and Analysis

The samples were compared with the biological controls for each batch and the following metrics were used to determine the QC metrics: scale factor, percent present, actin 3′ to 5′ ratio, GAPDH 3′ to 5′ ratio, and the RNA degradation slope. Affymetrix CEL files were processed using the BioConductor rma package [12], as well as the mas5 package for background correction and data normalization. No significant differences were found between these two data processing packages (data not shown), so the mas5 method was used for subsequent data analysis.

### 2.8. Repeatability and Reproducibility

To assess reproducibility, for each sample, RNA was extracted by a single user on three different days in Laboratory 1, and the same procedure was repeated by an independent user at Laboratory 2, resulting in 6 technical replicates for one biological sample. An intra-assay variability of less than 10% was expected when comparing the normalized results of the technical replicates in a single run. A review of the correlation between the counts of the technical replicates was prepared, with expected *R* ^2^correlation ≥ 0.98.

Reproducibility is the assessment of the same samples run by different users, on different days, and with different reagent lots to verify that there are minimal differences with respect to sample results. An interassay precision of less than 15% was expected when comparing the normalized results of the technical replicates between users, days, and reagents. A review of the correlation between the counts of the technical replicates was also prepared, with *R* ^2^ expected correlation ≥ 0.98.

### 2.9. Correlation

The samples were compared between platforms to assess the correlation of gene expression for common targets. We compared the Affymetrix gene expression data with NanoString expression data by normalizing the expression levels within each platform.

## 3. Results and Discussion

### 3.1. Testing Reproducibility with 60 Replicate Samples Using NanoString Platform and Affymetrix Microarray for mRNA Expression Measurement

Total RNA was extracted from 10 unique breast cancer FFPE samples (five TNB, and five ER+), respectively, in three different days by two different staff members, in two independent laboratories (Laboratory 1 and Laboratory 2), resulting in a total of 60 replicate samples. To achieve a more comprehensive view of the reproducibility in gene expression measurements, we employed cluster analysis, correlation analysis, and principal component analysis (PCA) to test if there were systematic biases among the samples processed differently.

**Figure 1:**
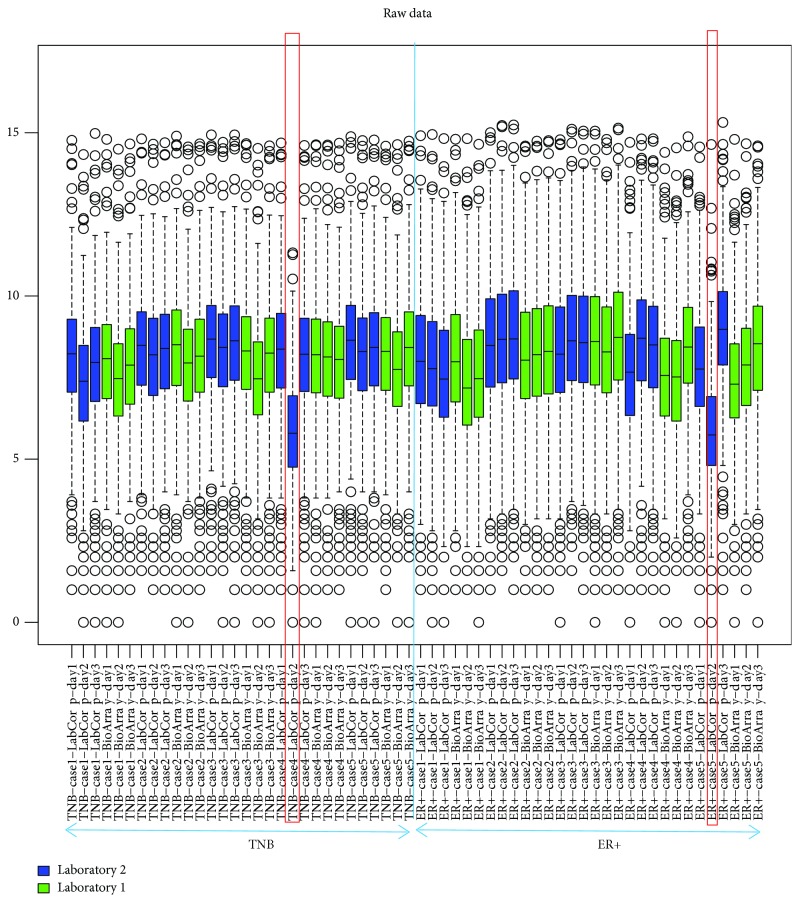
Boxplot analysis of the 60 replicate samples from NanoString experiment, with 5 unique TNB samples and 5 ER+ samples, prepared by two different labs and 3 different days. TNB: triple-negative breast cancer samples; ER+: estrogen receptor-positive breast cancer samples. The red lines show the outliers.

As the first step towards QC assessment of the data, boxplots with data before normalization were made to test if there were any outliers that were significantly different from the other samples tested. As shown in Figure 1, before normalization, the 60 samples showed a similar distribution of expression values, but two outliers were found to have notably different data point distributions in the boxplot. These results were subsequently confirmed with the normalized data (Supplementary 5). One of the outliers was the TNB case #4 sample prepared by Laboratory 2 at day 2, and the other one was the ER+ case #5 sample prepared by Laboratory 2 at day 2. When comparing the boxplot result to the RNA QC report, the two outliers identified from boxplot indeed failed the RNA mass cutoff.

To have a statistical evaluation of variation due to different biological samples vs. variation due to a system's random noise, we employed a mixed-model two-way ANOVA to test if the small variations among the 5 biological samples are indeed too minor to be detected by the NanoString platform (Supplementary 6). As a positive control, we first plotted the mean square (MS) of 5 ER+ biological samples vs. residual MS (system's random noise). As expected, the MS from the 5 ER+ samples was much bigger than residual MS, as evident in the plot that most of the data points are above the diagonal line (Supplementary 6), consistent with all the previous evidence that the 5 ER+ samples were statistically different. However, when a similar plot was made with MS of 5 TNB biological samples vs. residual MS, the data points are evenly distributed along both sides of the diagonal line (Supplementary 6), strongly suggesting that the variation among the 5 TNB samples are close to the system's random noise level.

Affymetrix CEL files were processed using the BioConductor mas5 package for background correction and data normalization. The quality of all the 60 samples from the Affymetrix experiment was assessed using BioConductor Affy package with histogram and boxplot as well as RNA degradation plot, and no sample was found to be significantly different from others (data not shown). The Affymetrix results from the 30 ER+ samples (Supplementary 7) were once again very consistent with the results obtained from the NanoString experiment. Two distinct clusters were formed among the 30 ER+ samples, where one cluster was composed of the 18 samples from ER+ cases 1-3 and another cluster was composed of the 12 samples from ER+ cases 4-5, exactly the same as what we observed in the NanoString experiment. PCA was used to further confirm the results from Supplementary 7. The PCA plots showed that the sample from TNB case #4 prepared by Laboratory 2 at day 2 is noticeably deviated from all the other TNB samples (Supplementary 8). The rest of the 29 TNB samples tended to form a large cluster, indicating that these samples were similar overall and could not be further separated. The PCA results for the 30 ER+ samples were notably different from the TNB PCA result but very similar to what was observed in the NanoString experiment. The cluster with all the case 1-3 samples was clearly different from the cluster with all the case 4-5 samples (Supplementary 8). In addition, it appeared that case 4-5 samples could be separated into two additional subclusters, but no additional subclusters were detected in the case 1-3 samples. No obvious laboratory effect was observed since the circles and squares were largely mingled with each other.

**Figure 2:**
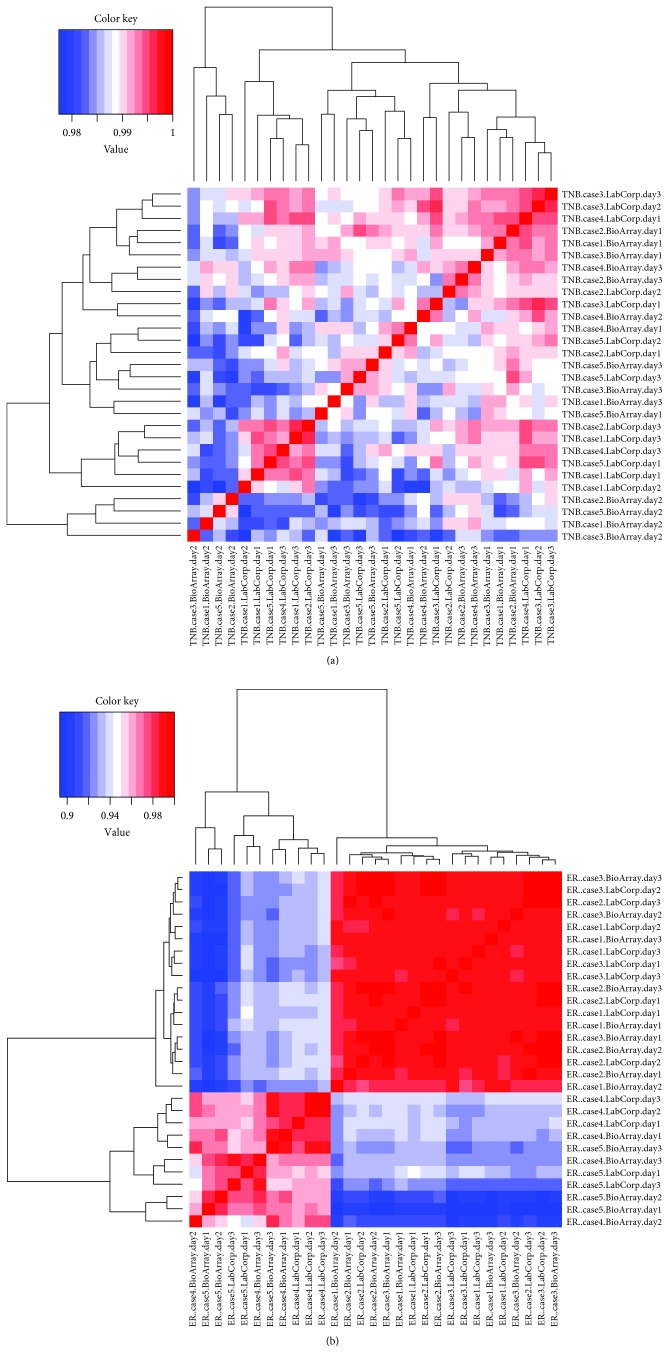
Heatmap view of the pairwise Pearson correlation coefficients among the 29 TNB and 29 ER+ samples. (a) Heatmap view of the pairwise Pearson correlation coefficients among the 29 TNB samples. (b) Heatmap view of the pairwise Pearson correlation coefficients among the 29 ER+ samples.

We calculated pairwise Pearson correlation coefficients among the 29 TNB samples and 29 ER+ samples that passed NanoString nCounter QC and visualized the data in the heatmap view (Figures 2(a) and 2(b)). As demonstrated by the correlation heatmap, there was very high correlation among all the 29 TNB samples, with the correlation coefficients ranging from 0.98 to 0.99 suggesting a similar expression pattern for all samples analyzed (Figure 2(a)). Considering the reproducibility of the samples and the NanoString platform, Veldman-Jones et al. [13] analyzing the expression of 6 replicates from different diffuse large B cell lymphoma (DLBCL) cell lines, the authors have found a Pearson value similar to the one found here of 0.981. Similarly, Chen et al. [14] using FFPE technical replicates from colon cancer specimens showed high reproducibility, with Pearson value of 0.983.

The correlation heatmap for the 29 ER+ samples further validated the finding from cluster and PCA (data not shown) that the samples from cases 1-3 (the red block on the top right) (Figure 2(b)) were different from those of cases 4-5 (the red block from bottom left) (Figure 2(b)), as visualized by the two blue blocks from the top left and bottom right, and the average correlation coefficient is 0.9 versus 0.98 within the case 1-3 samples or the case 4-5 samples. Gyanchandani et al. [15] have demonstrated that intratumoral heterogeneity is responsible for grouping tumors of the same molecular classification into different subgroups. Further, more recent studies have suggested that there are more subgroups among ER+ tumors, demonstrating that luminal A and B may not represent the majority of these tumors [16, 17].

**Figure 3:**
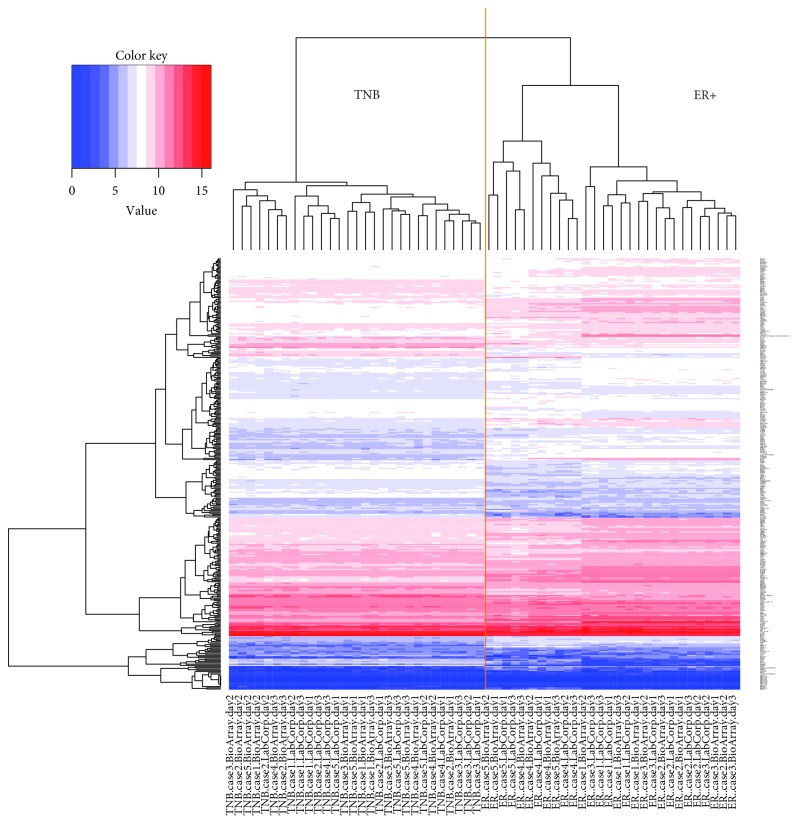
Heatmap view of the cluster analysis of the NanoString data from the 60 TNB and ER+ samples.

A large number of publications have shown that the TNB breast cancer subtype is significantly different from the ER+ breast cancer subtype at both the pathological level and the molecular level [18, 19]. For example, the TNB cancer may include molecular subtypes which include two basal-like, an immunomodulatory, a mesenchymal, a mesenchymal stem-like, and a luminal AR subtype [19]. Thus, we anticipated that TNB and ER+ samples should form two distinct clusters in an unsupervised cluster analysis. As shown in Figure 3, unsupervised clustering displayed two main clusters representing all 30 TNB samples in the left cluster and all 30 ER+ samples in the right cluster, confirming that there is a significant difference in the expression profiles of these two breast cancer subtypes.

**Figure 4:**
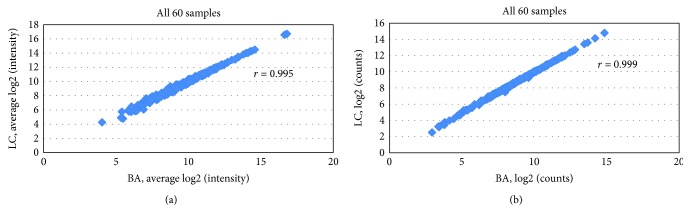
Scatter plot of the log2 expression values from Affymetrix and NanoString experiments by two laboratories. (a) Pearson correlation between sites Bioarray Laboratory (BA) and LabCorp Laboratory (LC) for all 60 samples using Affymetrix platform. (b) Pearson correlation between sites Bioarray Laboratory (BA) and LabCorp Laboratory (LC) for all 60 samples using NanoString platform.

To confirm if there is a significant difference between the results with RNA generated in the two different laboratories, we used scatter plots to compare the log2 count expression values for all 60 replicate samples. As shown, the Pearson correlation coefficients from the comparison were *R* = 0.995 for Affymetrix (Figure 4(a)) and *R* = 0.999 for NanoString nCounter (Figure 4(b)), respectively, indicating that the results from two laboratories are highly similar. To further investigate differences between the results with RNA generated in the 3 different days by laboratory, we used scatter plots to compare the log2 count expression values for all replicate samples. As shown in Supplementary 9, the range Pearson correlation coefficients from the comparisons were *R* = 0.987-0.988 and *R* = 0.982-0.991 for Affymetrix results from Laboratories 1 and 2, respectively. Supplementary 9 shows the range Pearson correlation coefficients *R* = 0.998-0.999 and *R* = 0.962-0.998 from the comparisons for NanoString gene expression for Laboratories 1 and 2, respectively. The results confirm that there are minimal variations between samples processed in two different laboratories in three different days by two independent users per gene expression counts in both Affymetrix and NanoString nCounter technologies.

### 3.2. Comparison between the Affymetrix Data and the NanoString Data

To compare the data from Affymetrix Human Genome U133 Plus 2.0 GeneChip® microarray to the NanoString data, we have mapped 325 endogenous genes and 7 housekeeping genes in the NanoString expression panel to all the potential probe sets in the U133 Plus 2.0 Array. The positive control and negative control genes present in the NanoString panel were excluded from the comparison.

As the first step towards comparing the two gene expression platforms, we examined whether a high expression level detected by NanoString for a particular gene of interest would show a similar high expression level when detected by the Affymetrix platform. We sorted the 332 genes present in the NanoString panel based on their average expression levels across the 12 samples (6 technical replicates from 1 TNB sample and 6 technical replicates from 1 ER+ sample). We then extracted the corresponding expression values for the sorted genes from the Affymetrix experiment, aligned them together, and viewed the aligned data in a heatmap view (data not shown).

We also calculated the pairwise Pearson correlation coefficients among the samples measured by the NanoString experiment vs. samples measured by the Affymetrix experiment. While the correlation among samples within the sample platform was quite high (range from 0.90 to 0.99), the correlation among samples between the two different platforms was poor (range from 0.4 to 0.5). From the RNA samples of DLBCL and using NanoString platform, Veldman-Jones et al. [13] have found a Pearson value of 0.954, when the correlation was done between freshly prepared RNA and FFPE-derived RNA (pair samples). However, unlike the samples analyzed here, when comparing NanoString and Affymetrix data, the analysis of the genetic expression from 34 DLBCL fresh samples, the Pearson value of 0.993 was found. But in another study [18], the correlation of expression between platforms was poor as demonstrated here, range from 0.63.

**Figure 5:**
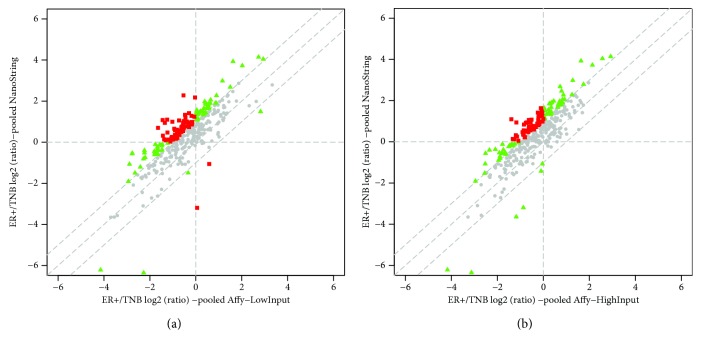
Scatter plots demonstrating the agreement and disagreement between the two platforms used. (a) Scatter plot of the ER+ signal/TNB signal ratio from Affymetrix (50 ng total RNA input) experiment vs. ER+/TNB ratio from NanoString experiment. (b) Scatter plot of the ER+ signal/TNB signal ratio from Affymetrix (100 ng total RNA input) experiment vs. ER+/TNB ratio from NanoString experiment.

One potential issue of directly comparing expression levels from two different platforms is whether or not a gene's endogenous expression level always correlates with the signal intensities measured by the two platforms. Woo et al. [20] suggested that for Affymetrix GeneChip® microarray, this might not be the case due to the probe design and hybridization chemistry. Thus, it is worth testing if changes in mRNA expression under different conditions for the same genes, not the absolute expression levels, are consistent between the two platforms. Since there were no control samples in the NanoString experiment, the ratio between the mean expression values from the 6 ER+ technical replicates over the mean values from the 6 TNB samples for all the 346 gene which are present in the NanoString and Affymetrix experiments was calculated. A moderate correlation was observed this time between the NanoString data and the Affymetrix data with both low and high RNA input, with Pearson correlation coefficients equal to 0.812 and 0.836, respectively. We have further tested the correlation of mRNA expression between the two platforms with the genes having the low, medium, and high expression levels. With the low RNA input, the correlation coefficients were 0.7565, 0.8540, and 0.8914 for low, medium, and high expression genes, respectively. With the high RNA input, the correlation coefficients were 0.8376, 0.839, and 0.9036 for low, medium, and high expression genes, respectively. Thus, genes with higher expression levels tend to have better correlation between the two platforms. In addition, higher RNA input also tends to improve the correlation between the two platforms. We defined concordant genes to be ones that have changes less than 2-fold (abs[log2(TNB/ER+)] < 1) (gray dots in Figure 5), plus the ones that have changes greater than 2-fold, but in the same direction from both platforms (green dots in Figures 5(a) and 5(b)). The discordant genes were defined as the ones which have opposite direction of changes (TNB vs. ER+) from the NanoString and Affymetrix experiments with magnitude of fold changes greater than 2-fold (red in Figures 5(a) and 5(b)). By this definition, 70-80% genes fell into the concordant category. It is important to point out that the correlation observed from this comparison is quite consistent with a number of published studies, demonstrating that the correlation between the NanoString and Affymetrix data was moderate [6, 9, 13–15, 21].

As already been discussed, a direct comparison between absolute signal intensities did not yield a good correlation between the gene expression measurements with the Affymetrix platform and those with the NanoString platform [6, 9, 13, 14, 21]. To test if we can make a similar observation with completely different samples, we randomly picked two additional cases, case #2 ER+ vs. case #2 TNB and case #4 ER+ vs. case #4 TNB, and repeated the analysis to test the concordance between the two platforms. In case there was a significant difference in the samples prepared by different laboratories, we analyzed the two laboratories independently. We calculated the ratio between the mean expression values from the 3 ER+ technical replicates from one particular lab over the mean values of the same lab's 3 TNB samples for all the 332 genes that are present in both the NanoString and Affymetrix experiments. The log2 ratios were then plotted and the results are shown in Supplementary 10 (case #2 and case #4). In case #2, a moderate correlation was observed once again between the NanoString data and the Affymetrix data. The Pearson correlation coefficient was 0.74 for the samples prepared by Laboratory 1 and is slightly better, 0.78, for the samples prepared by Laboratory 2. For case #4, the correlation appears to be mildly worse than case #2. The Pearson correlation coefficients were 0.68 and 0.64, for samples prepared by Laboratory 1 and Laboratory 2, respectively. It is important to point out that PCA strongly suggested that the sample from case #4 prepared by Laboratory 2 at day 2 could potentially be an outlier. This sample could possibly have accounted for the lower correlation observed in Supplementary 10.

We tested whether the correlation between the two platforms can be further improved with a larger sample size. The analysis was still done independently for the two different laboratories. This time, the ratio was done between the mean expression values from the 15 ER+ samples from one particular lab (5 cases all together) over the mean values from the 15 TNB samples from the same lab for all the 332 genes which are present in the NanoString and Affymetrix experiments. The log2 ratios were then plotted and the correlation between the two platforms had significantly improved when pooling a larger number of samples together. The Pearson correlation coefficients were 0.81 and 0.83, for samples prepared by Laboratory 1 and Laboratory 2, respectively.

**Figure 6:**
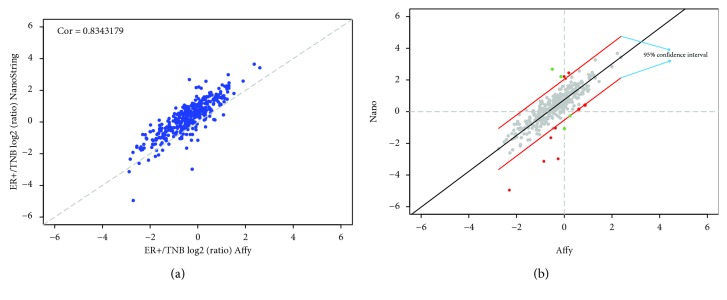
Scatter plot and linear regression from the samples comparing Affymetrix and NanoString results. (a) Scatter plot of the ER+ signal/TNB signal ratio from Affymetrix (50 ng total RNA input) experiment vs. ER+/TNB ratio from NanoString experiment. Samples were pooled from all the cases and different labs. (b) Linear regression of the scatter plots from Figure 6(a). The 2 red lines delineate the 95% confidence interval.

Finally, we took the ratio between the mean expression values from all the 30 ER+ samples (5 cases, with the 2 different labs combined) over the mean values from all the 30 TNB samples for all the 332 genes which are present in the NanoString and Affymetrix experiments. The log2 ratios were then plotted and the results are shown in Figure 6. As we can see, pooling all the 30 samples for a particular disease type further improved, but at a marginal level, the correlation over the ones we obtained when pooling 15 samples prepared by Laboratory 2. In Figure 6(a), we also noticed that there is an overall higher ER+/TNB ratio detected by NanoString than by Affymetrix, and the data points were not distributed evenly along the (0, 1) diagonal line. To more accurately identify genes that show concordant vs. discordant expression between the Affymetrix platform and the NanoString platform, linear regression (lm) was applied and a regression line was drawn to help define the data points to be concordant or discordant (Figure 6(b)).

We then defined the concordant genes to be the ones that have changes less than lm predicted values ± 2 standard deviation at 95% confidence interval (gray dots in Figure 6(b)), plus the ones that have changes greater than lm predicted values ± 2 standard deviation, but in the same direction from both platforms (red dots in Figure 6(b)). The discordant genes were defined as the ones which have opposite direction of changes (TNB vs. ER+) from the NanoString and Affymetrix experiments with changes greater than lm predicted values ± 2 standard deviation (green in Figure 6(b)). By this definition, almost 90% genes fell into the concordant category (Figure 6(b)), as opposed to the 70-80% when only 1 case was used. Only 4 genes displayed discordant changes between the two platforms, further confirming that pooling larger number of samples together significantly improves the consistency between the two platforms.

## 4. Conclusions

The reproducibility among the technical replicates for both the NanoString platform and the Affymetrix platform was high, as demonstrated by heatmaps, cluster analyses, and correlation analysis. A high similarity was observed among the 5 unique TNB samples included in this study using the genes we examined, as shown by all 6 technical replicates for all 5 unique samples forming a single cluster. The ER+ samples presented a differential expression profile for two samples, but the replication grouping was the same, demonstrating that intratumoral heterogeneity could exist between tumors of the same molecular subtype. Although the correlation between NanoString and Affymetrix platforms was not good when directly comparing absolute signal intensities, there appears a moderate concordance between these two platforms when using changes in gene expression. Further, the concordance can be improved significantly when pooling a larger number of samples. We should consider that the degree of degradation of RNA derived from FFPE samples is still a challenge for transcriptome analysis, and further studies should be conducted for comparison between samples and expression analysis platforms.

## Data Availability

The data used to support the findings of this study are available from the corresponding author upon request.

## Abbreviations

FFPE: Formalin-fixed paraffin embedded
QC: Quantification and quality
TNB: Triple-negative breast cancer
ER+: Estrogen receptor-positive
UHR: Universal human reference
DLBCL: Diffuse large B cell lymphoma.

## Supplementary Materials

Supplementary 1 Supplementary Table 1: description of the cases analyzed. Table 1a: TNB cases. Table 1b: ER+ negative cases.

Supplementary 2 Supplementary Table 2: samples QC information.

Supplementary 3 Supplementary Table 3: list of genes.

Supplementary 4 Supplementary Table 4: nCounter™ performance QC metrics.

Supplementary 5 Supplementary Figure 1: heatmap view of the normalized NanoString data from 30 ER+ and 30 TNB samples. a: TNB cases. TNB case #4 sample identified through boxplot analysis prepared by Laboratory 2 at day 2 was noteworthy different from the rest of the 29 samples. b: ER+ cases. The separation demonstrates cases 4-5 with different pattern of expression and case 5 analyzed at Laboratory 2 at day 2showing quite significant deviation.

Supplementary 6 Supplementary Figure 2: ANOVA to test if the small variations among the biological samples are indeed too minor to be detected by the NanoString platform. a: two-way ANOVA of the MS comparison among the 346 genes from the 29 ER+ samples. b: two-way ANOVA of the MS comparison among the 346 genes from the 29 TNB samples.

Supplementary 7 Supplementary Figure 3: heatmaps of the TNB and ER samples using the Affymetrix platform. a: heatmap view of the cluster analysis of the 30 TNB samples. b: heatmap view of the cluster analysis of the 30 ER+ samples.

Supplementary 8 Supplementary Figure 4: PCA of the 30 TNB samples (a) and the 30 ER+ samples (b).

Supplementary 9 Supplementary Figure 5: scatter plots generated in the 3 different days by laboratory to compare the log2 count expression values for all replicate samples. a-b: the range Pearson correlation coefficients from the comparisons were *R* = 0.987-0.988 and *R* = 0.982-0.991 for Affymetrix results from Laboratories 1 and 2, respectively. c-d: the range Pearson correlation coefficients *R* = 0.998-0.999 and *R* = 0.962-0.998 from the comparisons for NanoString gene expression for Laboratories 1 and 2, respectively.

Supplementary 10 Supplementary Figure 6: scatters plots comparing mRNA measurement using Affymetrix and NanoString platforms. a: scatter plot of the ER+ signal/TNB signal ratio from Affymetrix (50 ng total RNA input) experiment vs. ER+/TNB ratio from NanoString experiment. Samples were from case 2 and prepared by Laboratory 1. b: scatter plot of the ER+ signal/TNB signal ratio from Affymetrix (100 ng total RNA input) experiment vs. ER+/TNB ratio from NanoString experiment. Samples were from case 2 and prepared by Laboratory 2. c: scatter plot of the ER+ signal/TNB signal ratio from Affymetrix (50 ng total RNA input) experiment vs. ER+/TNB ratio from NanoString experiment. Samples were from case 4 and prepared by Laboratory 1. d: scatter plot of the ER+ signal/TNB signal ratio from Affymetrix (100 ng total RNA input) experiment vs. ER+/TNB ratio from NanoString experiment. Samples were from case 4 and prepared by Laboratory 2.
