# Supplementary material for: Expression Concordance of 325 Novel RNA Biomarkers between Data Generated by NanoString nCounter and Affymetrix GeneChip
Source: Dis Markers. 2019 May 14;2019:1940347. doi: 10.1155/2019/1940347 (PMC6536986; doi:10.1155/2019/1940347)
Supplement: Supplementary 1 — Supplementary Table 1: description of the cases analyzed. Table 1a: TNB cases. Table 1b: ER+ negative cases. [file 1940347.f1.docx]

Supplementary Table 1: Description of the cases analyzed.

Supplementary Table 1a. TNB cases.

|  | Case# 1 | Case# 2 | Case# 3 | Case# 4 | Case# 5 |
| --- | --- | --- | --- | --- | --- |
| Gender | F | F | F | F | F |
| Age | 70 | 68 | 78 | 72 | 65 |
| Date of Surgery | 1/13/2015 | 10/12/2014 | 6/25/2014 | 8/22/2014 | 2/13/2015 |
| Ethnicity | Caucasian | Caucasian | Caucasian | Caucasian | Caucasian |
| Sample Type | FFPE | FFPE | FFPE | FFPE | FFPE |
| Source | Left Breast | Right Breast | Right Breast | Left Breast | Left Breast |
| Diagnosis | Infiltrating ductal carcinoma | | | | |
| Tumor content% | 60% | 95% | 60% | 65% | 80% |
| Grade | G3 | G2 | G2 | G2 | G3 |
| Stage | IIB | IIA | IIA | IIA | IIB |
| HER2 NEU | 0 | 1+ | 0 | 0 | 0 |
| ER | Negative | Negative | Negative | Negative | Negative |
| PR | Negative | Negative | Negative | Negative | Negative |
| Ki-67 | 10 | 20 | 10 | 10 | 20 |
| Tumor size | 5.0 x 2.0 x 3.0 | 3.0 x 3.0 x 2.0 | 3.5 x 2.0 x 2.0 | 3.0 x 4.0 x 4.0 | 3.5 x 4.5 x 3.5 |
| Metastases | 2/7 lymph nodes | 0/9 lymph nodes | 0/9 lymph nodes | 0/9 lymph nodes | 2/7 lymph nodes |
| TNM | T2N1M0 | T2N0M0 | T2N0M0 | T2N0M0 | T2N1M0 |
| Diagnosis | Cancer | Cancer | Cancer | Cancer | Cancer |

|  | Case# 1 | Case# 2 | Case# 3 | Case# 4 | Case# 5 |
| --- | --- | --- | --- | --- | --- |
| Gender | F | F | F | F | F |
| Age | 69 | 65 | 59 | 72 | 75 |
| Date of Surgery | 1/21/2015 | 8/9/2014 | 2/26/2015 | 3/5/2015 | 9/13/2014 |
| Ethnicity | Caucasian | Caucasian | African American | Caucasian | Caucasian |
| Sample Type | FFPE | FFPE | FFPE | FFPE | FFPE |
| Source | Right Breast | Left Breast | Right Breast | Right Breast | Left Breast |
| Diagnosis | Infiltrating ductal carcinoma | | | | |
| Tumor content% | 70% | 80% | 75% | 80% | 95% |
| Grade | G2 | G2 | G3 | G2 | G3 |
| Stage | IIA | IIB | IIIA | IIA | IIIB |
| HER2 NEU | 0 | 0 | +1 | 0 | 0 |
| ER | Positive | Positive | Positive | Positive | Positive |
| PR | Negative | Negative | Negative | Negative | Negative |
| Ki-67 | 20 | 30 | 30 | 40 | 30 |
| Tumor size | 4.5 x 3.0 x 3.5 | 1.8 x 2.5 x 2.8 | 2.5 x 4.6 x 3.5 | 5.2 x 3.0 x 2.4 | 3,0 x 2.5 x 3.6 |
| Metastases | 0/9 lymph nodes | 2/7 lymph nodes | 1/9 lymph nodes | 0/9 lymph nodes | 0/9 lymph nodes |
| TNM | T2N0M0 | T2N1M0 | T2N2M0 | T2N0M0 | T4N0M0 |
| Diagnosis | Cancer | Cancer | Cancer | Cancer | Cancer |

Supplementary Table 1b. ER+ cases.
