# Supplementary material for: Expression Concordance of 325 Novel RNA Biomarkers between Data Generated by NanoString nCounter and Affymetrix GeneChip
Source: Dis Markers. 2019 May 14;2019:1940347. doi: 10.1155/2019/1940347 (PMC6536986; doi:10.1155/2019/1940347)
Supplement: Supplementary 2 — Supplementary Table 2: samples QC information. [file 1940347.f2.docx]

Supplementary Table 2: Samples QC information

| BioSample Name | Curr Mass (µg) | QC Status | Conc. Status | Curr Vol (µL) | Curr Conc (µg/µL) | 28S/18S | RIN |
| --- | --- | --- | --- | --- | --- | --- | --- |
| ER+, day1, case1 | 7.25 | PASS | PASS | 39.30 | 0.185 | 0 | 2.07 |
| ER+, day1, case 2 | 5.681 | PASS | PASS | 36.80 | 0.154 | 0 | 1.93 |
| ER+, day1, case 3 | 7.53 | PASS | PASS | 34.40 | 0.219 | 0 | 2.12 |
| ER+, day1, case 4 | 0.624 | PASS | PASS | 41.90 | 0.015 | 0 | 2.32 |
| ER+, day1, case 5 | 0.375 | PASS | PASS | 43.20 | 0.009 | 6.612 | 2.74 |
| TNB, day1, case 1 | 7.17 | PASS | PASS | 60.00 | 0.120 | 0.082 | 2.33 |
| TNB, day1, case 2 | 15.969 | PASS | PASS | 71.10 | 0.225 | 0 | 2.29 |
| TNB, day1, case 3 | 13.468 | PASS | PASS | 72.80 | 0.185 | 0.637 | 2.47 |
| TNB, day1, case 4 | 13.919 | PASS | PASS | 72.80 | 0.191 | 0.169 | 2.25 |
| TNB, day1, case 5 | 15.542 | PASS | PASS | 72.80 | 0.214 | 0.635 | 2.49 |
| ER+, day2, case 1 | 8.09 | FAIL | FAIL | 41.90 | 0.193 | 0 | 1.98 |
| ER+, day2, case 2 | 9.132 | PASS | PASS | 43.20 | 0.211 | 0 | 1.97 |
| ER+, day2, case 3 | 8.811 | PASS | PASS | 41.90 | 0.210 | 0 | 1.99 |
| ER+, day2, case 4 | 0.425 | PASS | PASS | 45.80 | 0.009 | 0 | 2.27 |
| ER+, day2, case 5 | 0.481 | PASS | PASS | 47.20 | 0.010 | 0 | 2.22 |
| TNB, day2, case 1 | 9.349 | PASS | PASS | 76.20 | 0.123 | 0.113 | 2.28 |
| TNB, day2, case 2 | 9.934 | PASS | PASS | 40.60 | 0.245 | 0.09 | 2.26 |
| TNB, day2, case 3 | 7.722 | PASS | PASS | 41.90 | 0.184 | 0.1 | 2.37 |
| TNB, day2, case 4 | 7.95 | PASS | PASS | 39.30 | 0.202 | 8.857 | 2.91 |
| TNB, day2, case 5 | 6.771 | PASS | PASS | 41.90 | 0.162 | 0.818 | 2.35 |
| ER+, day3, case 1 | 9.558 | PASS | PASS | 45.80 | 0.209 | 0 | 1.98 |
| ER+, day3, case 2 | 9.91 | PASS | PASS | 50.00 | 0.198 | 0 | 1.98 |
| ER+, day3, case 3 | 8.398 | PASS | PASS | 48.60 | 0.173 | 0 | 2.33 |
| ER+, day3, case 4 | 0.494 | PASS | PASS | 17.90 | 0.028 | 0 | 2.50 |
| ER+, day3, case 5 | 1.119 | PASS | PASS | 22.00 | 0.051 | 0 | 2.25 |
| TNB, day3, case 1 | 14.969 | PASS | PASS | 110.80 | 0.135 | 0.783 | 2.50 |
| TNB, day3, case 2 | 11.037 | PASS | PASS | 43.20 | 0.256 | 0.395 | 2.36 |
| TNB, day3, case 3 | 17.969 | PASS | PASS | 47.20 | 0.381 | 5.021 | 3.42 |
| TNB, day3, case 4 | 11.028 | PASS | PASS | 43.20 | 0.255 | 5.506 | 3.59 |
| TNB, day3, case 5 | 13.81 | PASS | PASS | 47.20 | 0.293 | 0.375 | 2.41 |
| ER+ CASE#1 LC-1 | 4.767 | PASS | PASS | 47.20 | 0.101 | 0 | 2.08 |
| ER+ CASE#2 LC-1 | 6.799 | PASS | PASS | 48.60 | 0.140 | 0 | 2.04 |
| ER+ CASE#3 LC-1 | 7.207 | PASS | PASS | 47.20 | 0.153 | 0 | 1.99 |
| ER+ CASE#4 LC-1 | 0.699 | PASS | PASS | 48.60 | 0.014 | 0 | 2.09 |
| ER+ CASE#5 LC-1 | 0.495 | PASS | PASS | 48.60 | 0.010 | 0 | 2.36 |
| TNB CASE#1 LC-1 | 19.061 | PASS | PASS | 175.20 | 0.109 | 0 | 2.22 |
| TNB CASE#2 LC-1 | 13.451 | PASS | PASS | 110.80 | 0.121 | 0.118 | 2.31 |
| TNB CASE#3 LC-1 | 17.359 | PASS | PASS | 169.20 | 0.103 | 0 | 2.28 |
| TNB CASE#4 LC-1 | 15.7 | PASS | PASS | 146.60 | 0.107 | 0 | 2.40 |
| TNB CASE#5 LC-1 | 12.615 | PASS | PASS | 104.00 | 0.121 | 0 | 2.40 |
| ER+ CASE#1 LC-2 | 6.249 | PASS | PASS | 47.20 | 0.132 | 0 | 2.07 |
| ER+ CASE#2 LC-2 | 3.769 | PASS | PASS | 45.80 | 0.082 | 0 | 2.13 |
| ER+ CASE#3 LC-2 | 6.225 | PASS | PASS | 47.20 | 0.132 | 0 | 2.03 |
| ER+ CASE#4 LC-2 | 0.745 | PASS | PASS | 50.00 | 0.015 | 0 | 2.27 |
| ER+ CASE#5 LC-2 | 0.149 | PASS | PASS | 51.40 | 0.003 | 0 | 2.31 |
| TNB CASE#1 LC-2 | 13.583 | FAIL | FAIL | 113.10 | 0.120 | 7.274 | 3.12 |
| TNB CASE#2 LC-2 | 10.932 | PASS | PASS | 101.70 | 0.108 | 0 | 3.45 |
| TNB CASE#3 LC-2 | 8.809 | PASS | PASS | 91.10 | 0.097 | 0 | 2.30 |
| TNB CASE#4 LC-2 | 0.283 | PASS | PASS | 83.40 | 0.003 | 0 | 2.36 |
| TNB CASE#5 LC-2 | 10.7 | PASS | PASS | 89.10 | 0.120 | 8.57 | 3.62 |
| ER+ CASE#1 LC-3 | 5.083 | PASS | PASS | 45.80 | 0.111 | 0 | 2.16 |
| ER+ CASE#2 LC-3 | 8.057 | PASS | PASS | 41.90 | 0.192 | 0 | 2.04 |
| ER+ CASE#3 LC-3 | 7.97 | PASS | PASS | 48.60 | 0.164 | 0 | 2.12 |
| ER+ CASE#4 LC-3 | 0.873 | PASS | PASS | 47.20 | 0.019 | 0 | 2.22 |
| ER+ CASE#5 LC-3 | 0.575 | PASS | PASS | 51.40 | 0.011 | 0 | 2.58 |
| TNB CASE#1 LC-3 | 20.597 | PASS | PASS | 146.60 | 0.141 | 0 | 2.29 |
| TNB CASE#2 LC-3 | 16.53 | PASS | PASS | 166.30 | 0.099 | 0 | 2.25 |
| TNB CASE#3 LC-3 | 14.515 | PASS | PASS | 122.60 | 0.118 | 0 | 2.17 |
| TNB CASE#4 LC-3 | 10.55 | PASS | PASS | 81.60 | 0.129 | 3.653 | 2.49 |
| TNB CASE#5 LC-3 | 11.957 | PASS | PASS | 89.10 | 0.134 | 0 | 2.33 |

LC: LabCorp/Covance.
