# Supplementary material for: Expression Concordance of 325 Novel RNA Biomarkers between Data Generated by NanoString nCounter and Affymetrix GeneChip
Source: Dis Markers. 2019 May 14;2019:1940347. doi: 10.1155/2019/1940347 (PMC6536986; doi:10.1155/2019/1940347)
Supplement: Supplementary 4 — Supplementary Table 4: nCounter™ performance QC metrics. [file 1940347.f4.docx]

Supplementary Table 4: nCounter™ Performance QC-Metrics.

| **Performance QC-Metrics** | **Acceptance Criteria** |
| --- | --- |
| Imaging Field of View (FOV) | >0.75 |
| Binding density (number of reporters per µm^2^) | 0.05-2.25 |
| Positive control scaling factor (Normalization Factor) | 0.3-3.0 |
| Linear dynamic range | up to 7x10^5^ total counts |
| Positive control spike-in correlation | R^2^>0.95; Linearity >2.5 logs of expression range |
| Limit of Detection | 0.5fM spike-in control (~1 copy per cell); 90%  of the time |
| Coefficient of Variation (CV) in biological replicates | ≤ 25% |
| Coefficient of variation in technical replicates | ≤ 15% |
