# Supplementary material for: Expression Concordance of 325 Novel RNA Biomarkers between Data Generated by NanoString nCounter and Affymetrix GeneChip
Source: Dis Markers. 2019 May 14;2019:1940347. doi: 10.1155/2019/1940347 (PMC6536986; doi:10.1155/2019/1940347)
Supplement: Supplementary 5 — Supplementary Figure 1: heatmap view of the normalized NanoString data from 30 ER+ and 30 TNB samples. a: TNB cases. TNB case #4 sample identified through boxplot analysis prepared by Laboratory 2 at day 2 was noteworthy different from the rest of the 29 samples. b: ER+ cases. The separation demonstrates cases 4-5 with different pattern of expression and case 5 analyzed at Laboratory 2 at day 2showing quite significant deviation. [file 1940347.f5.docx]

Supplementary Figure 1: Heatmap view of the normalized NanoString data from 30 ER+ and 30 TNB samples.


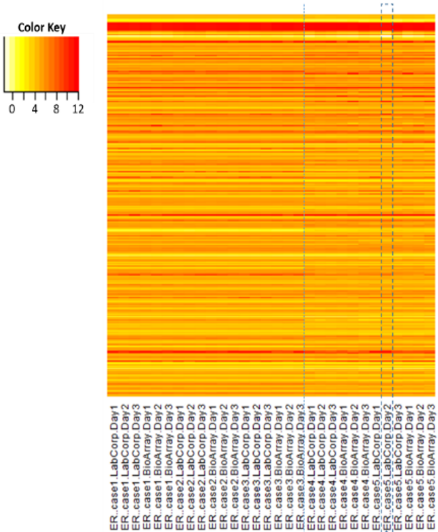

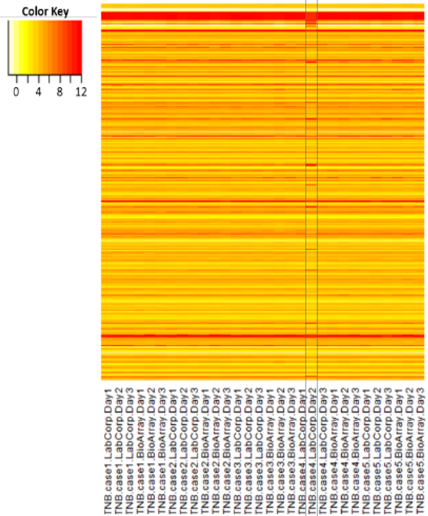


Supplementary Figure 1a: TNB cases. TNB case #4 sample identified through boxplot analysis, prepared by Laboratory 2 at day 2 was noteworthy different from the rest of the 29 samples.

Supplementary Figure 1b: ER+ cases. The separation demonstrates cases 4-5 with different pattern of expression, and case 5 analyzed at Laboratory 2 at day 2, showing quite significant deviation.
