# Supplementary material for: Expression Concordance of 325 Novel RNA Biomarkers between Data Generated by NanoString nCounter and Affymetrix GeneChip
Source: Dis Markers. 2019 May 14;2019:1940347. doi: 10.1155/2019/1940347 (PMC6536986; doi:10.1155/2019/1940347)
Supplement: Supplementary 6 — Supplementary Figure 2: ANOVA to test if the small variations among the biological samples are indeed too minor to be detected by the NanoString platform. a: two-way ANOVA of the MS comparison among the 346 genes from the 29 ER+ samples. b: two-way ANOVA of the MS comparison among the 346 genes from the 29 TNB samples. [file 1940347.f6.docx]

Supplementary Figure 2: ANOVA analysis to test if the small variations among the biological samples are indeed too minor to be detected by the NanoString platform.


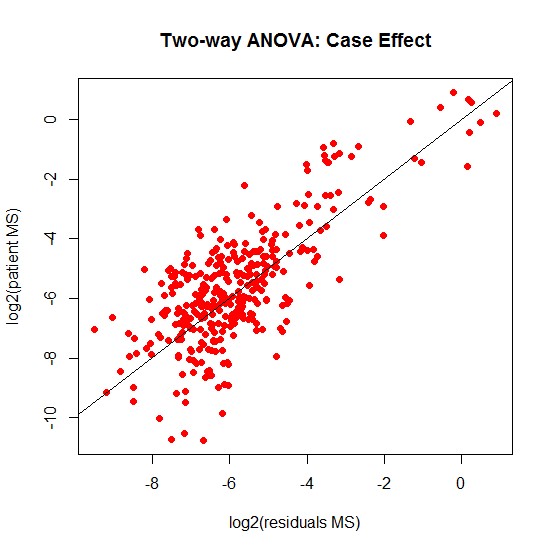

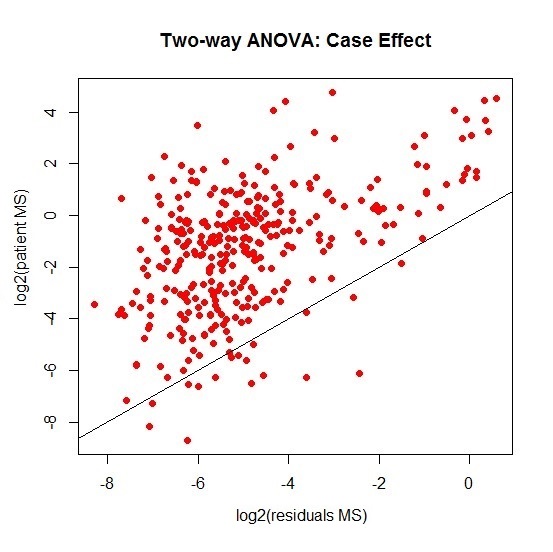


Supplementary Figure 2a: Two-way ANOVA analysis of the MS comparison among the 346 genes from the 29 ER+ samples.

Supplementary Figure 2b: Two-way ANOVA analysis of the MS comparison among the 346 genes from the 29 TNB samples.
