# Supplementary material for: Expression Concordance of 325 Novel RNA Biomarkers between Data Generated by NanoString nCounter and Affymetrix GeneChip
Source: Dis Markers. 2019 May 14;2019:1940347. doi: 10.1155/2019/1940347 (PMC6536986; doi:10.1155/2019/1940347)
Supplement: Supplementary 7 — Supplementary Figure 3: heatmaps of the TNB and ER samples using the Affymetrix platform. a: heatmap view of the cluster analysis of the 30 TNB samples. b: heatmap view of the cluster analysis of the 30 ER+ samples. [file 1940347.f7.docx]

| 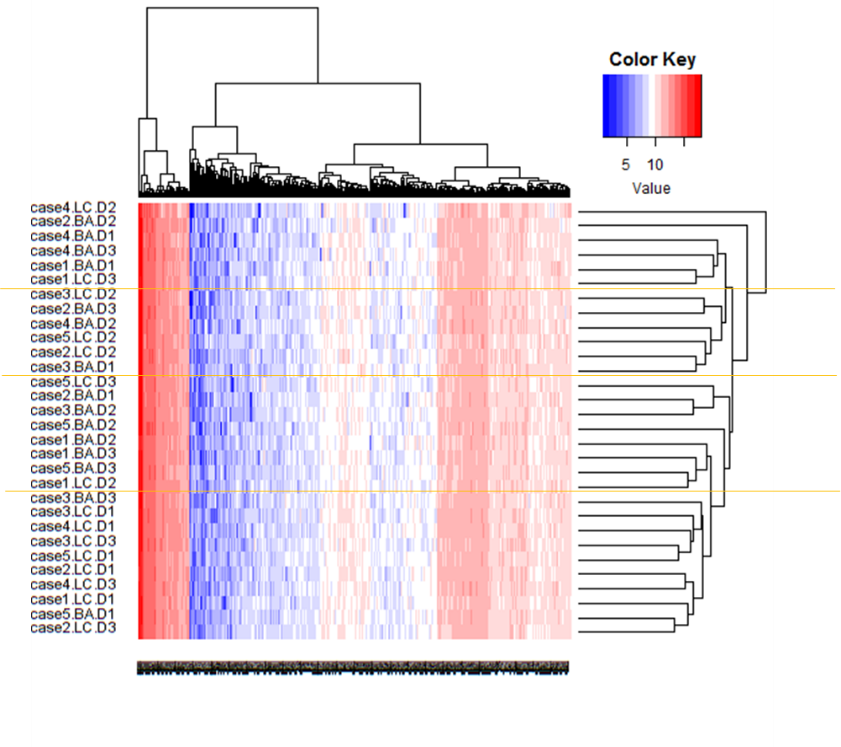 | 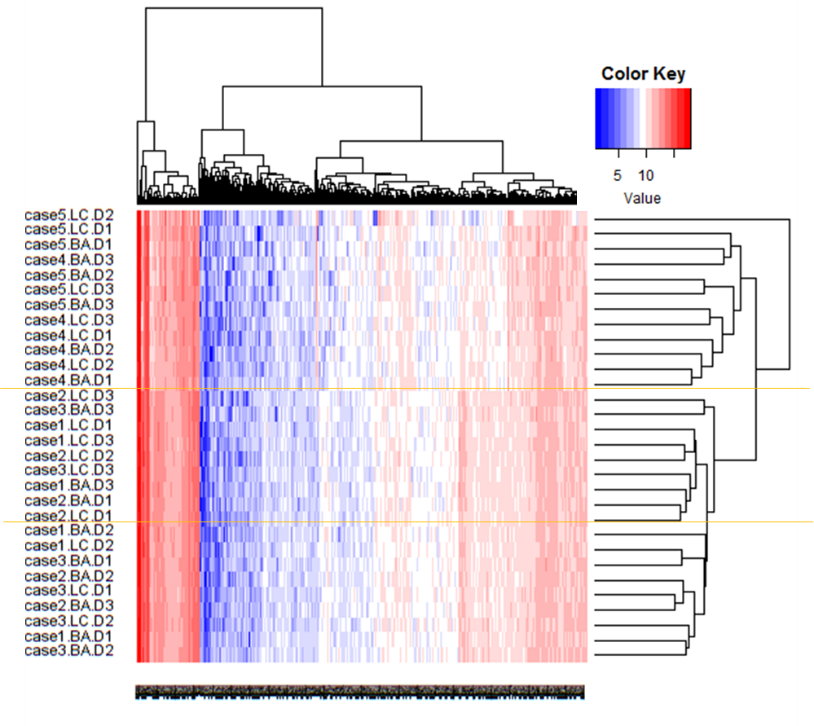 |
| --- | --- |
| Supplementary Figure 3a: Heatmap view of the cluster analysis of the 30 TNB samples. | Supplementary Figure 3b: Heatmap view of the cluster analysis of the 30 ER+ samples. |

Supplementary Figure 3: Heatmaps of the TNB and ER samples using the Affymetrix platform.

LC: LabCorp/Covance Laboratory; BA: Bioarray Laboratory.
