# Supplementary figures and images for: Expression Concordance of 325 Novel RNA Biomarkers between Data Generated by NanoString nCounter and Affymetrix GeneChip
Source: Dis Markers. 2019 May 14;2019:1940347. doi: 10.1155/2019/1940347 (PMC6536986; doi:10.1155/2019/1940347)

Supplementary Figure 4: PCA analysis of the 30 TNB samples (a) and the 30 ER+ samples (b).

| a)  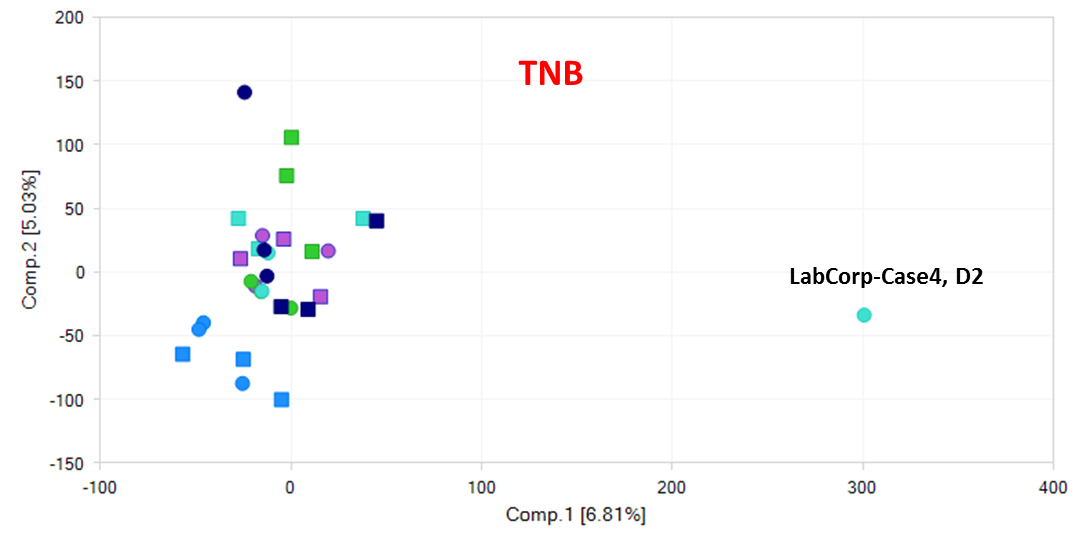 |  |
| --- | --- |
| b)  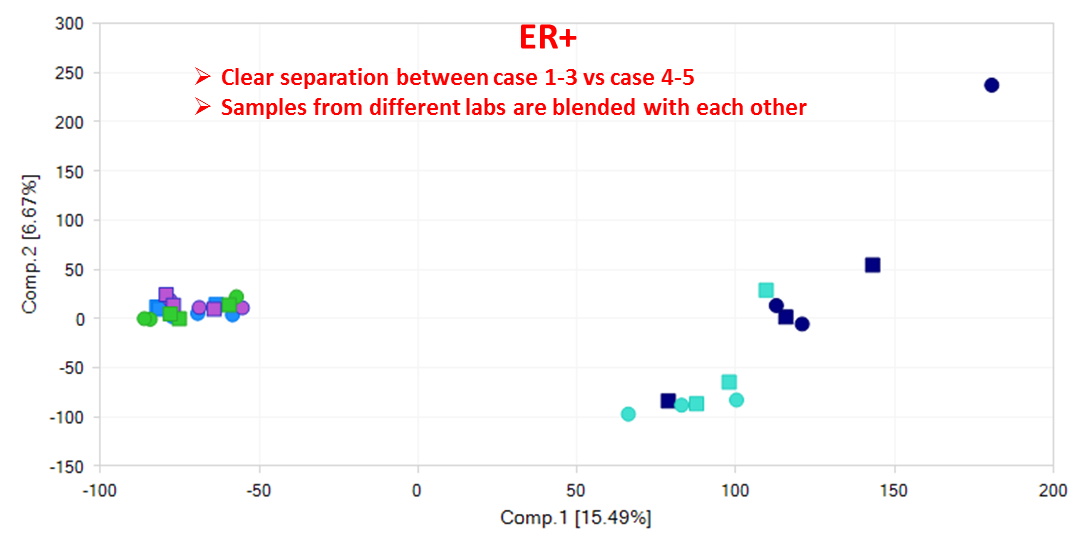 |

Supplement: Supplementary 8 — Supplementary Figure 4: PCA of the 30 TNB samples (a) and the 30 ER+ samples (b). [file 1940347.f8.docx]
