# Supplementary material for: Expression Concordance of 325 Novel RNA Biomarkers between Data Generated by NanoString nCounter and Affymetrix GeneChip
Source: Dis Markers. 2019 May 14;2019:1940347. doi: 10.1155/2019/1940347 (PMC6536986; doi:10.1155/2019/1940347)
Supplement: Supplementary 9 — Supplementary Figure 5: scatter plots generated in the 3 different days by laboratory to compare the log2 count expression values for all replicate samples. a-b: the range Pearson correlation coefficients from the comparisons were R = 0.987-0.988 and R = 0.982-0.991 for Affymetrix results from Laboratories 1 and 2, respectively. c-d: the range Pearson correlation coefficients R = 0.998-0.999 and R = 0.962-0.998 from the comparisons for NanoString gene expression for Laboratories 1 and 2, respectively. [file 1940347.f9.docx]

Supplementary Figure 5: Scatter plots generated in the 3 different days by laboratory to compare the Log2 counts expression values for all replicate samples.


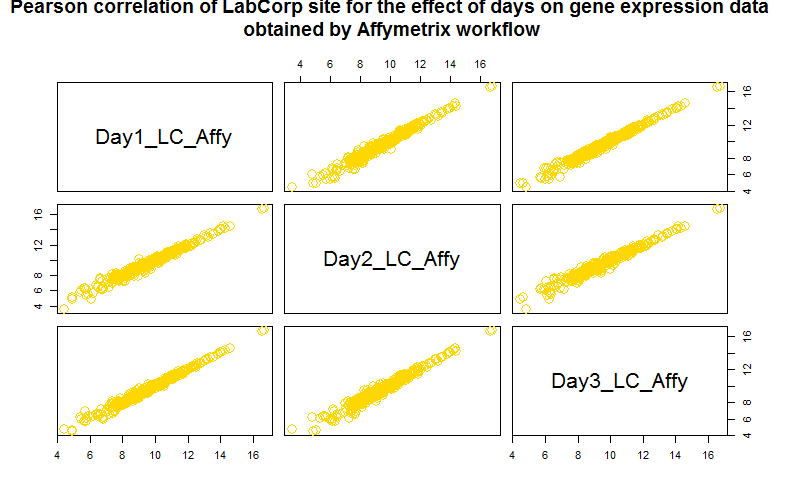


R=0.987

R=0.991

R=0.982

R=0.987

R=0.982

R=0.991

a)


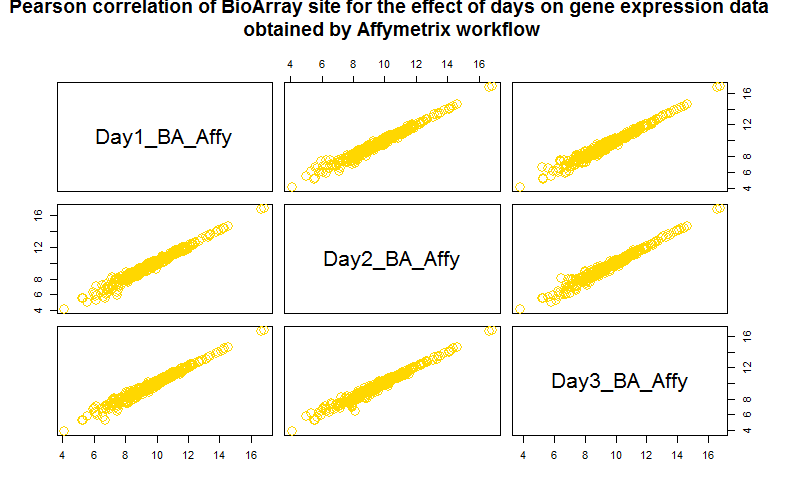


R=0.987

R=0.988

R=0.987

R=0.988

R=0.987

R=0.987

b)

Supplementary Figure 5 a-b: The range Pearson correlation coefficiencies from the comparisons were R=0.987-0.988 and R=0.982-0.991 for Affymetrix results from Laboratory 1 and 2, respectively.


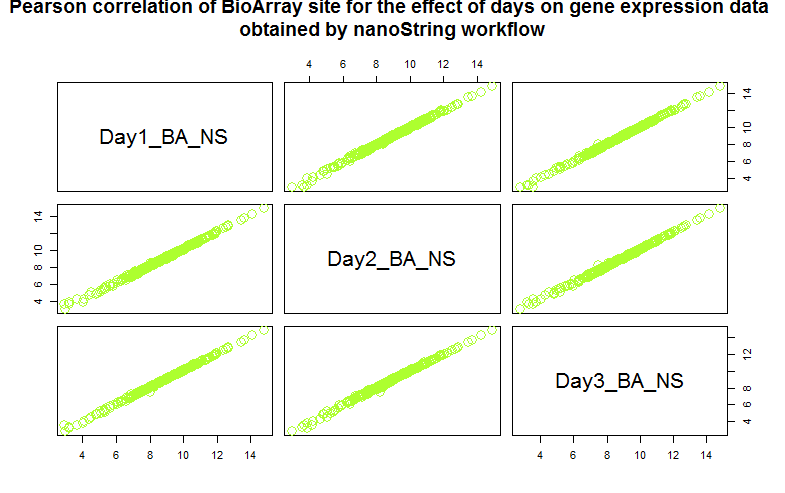


R=0.998

R=0.998

R=0.999

R=0.998

R=0.998

R=0.999

c)


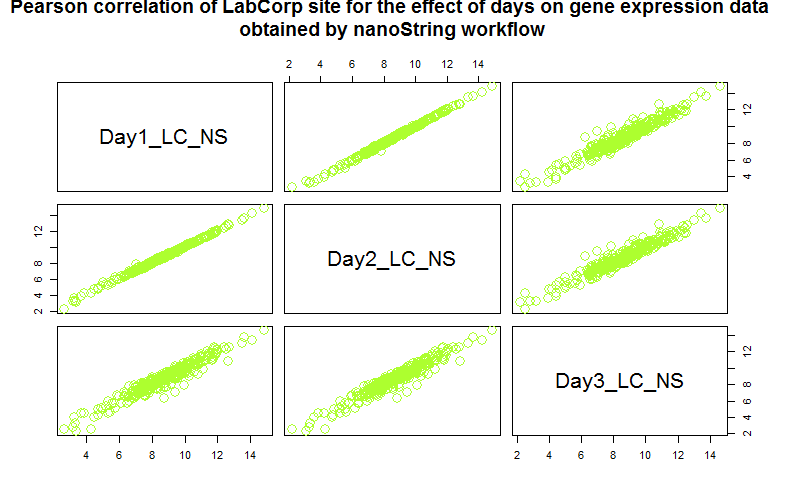


R=0.998

R=0.962

R=0.963

R=0.998

R=0.962

R=0.963

d)

Supplementary Figure 5 c-d: The range Pearson correlation coefficiencies R=0.998-0.999 and R=0.962-0.998 from the comparisons for NanoString gene expression for Laboratory 1 and 2, respectively.
