# Supplementary material for: Expression Concordance of 325 Novel RNA Biomarkers between Data Generated by NanoString nCounter and Affymetrix GeneChip
Source: Dis Markers. 2019 May 14;2019:1940347. doi: 10.1155/2019/1940347 (PMC6536986; doi:10.1155/2019/1940347)
Supplement: Supplementary 10 — Supplementary Figure 6: scatters plots comparing mRNA measurement using Affymetrix and NanoString platforms. a: scatter plot of the ER+ signal/TNB signal ratio from Affymetrix (50 ng total RNA input) experiment vs. ER+/TNB ratio from NanoString experiment. Samples were from case 2 and prepared by Laboratory 1. b: scatter plot of the ER+ signal/TNB signal ratio from Affymetrix (100 ng total RNA input) experiment vs. ER+/TNB ratio from NanoString experiment. Samples were from case 2 and prepared by Laboratory 2. c: scatter plot of the ER+ signal/TNB signal ratio from Affymetrix (50 ng total RNA input) experiment vs. ER+/TNB ratio from NanoString experiment. Samples were from case 4 and prepared by Laboratory 1. d: scatter plot of the ER+ signal/TNB signal ratio from Affymetrix (100 ng total RNA input) experiment vs. ER+/TNB ratio from NanoString experiment. Samples were from case 4 and prepared by Laboratory 2. [file 1940347.f10.docx]

| a)  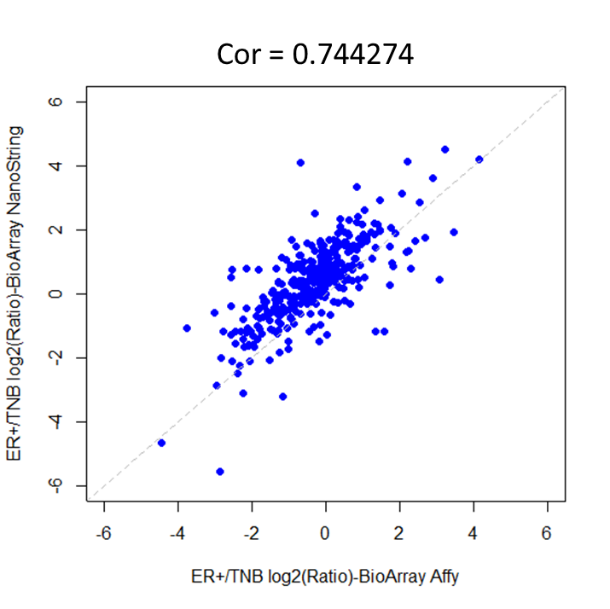 | b)  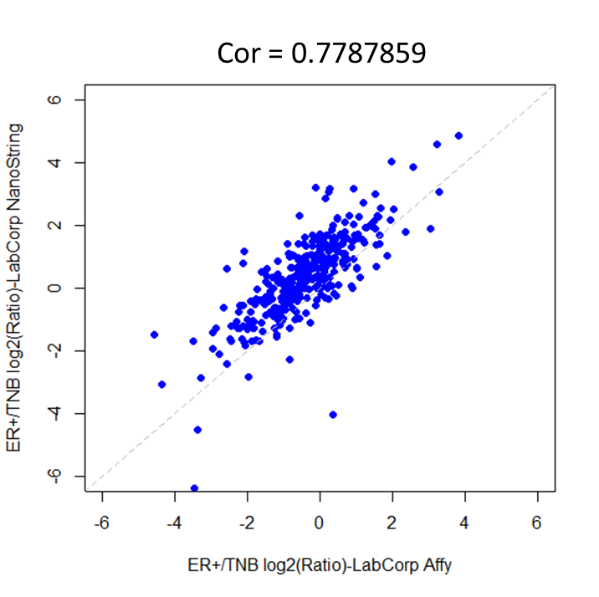 |
| --- | --- |
| Supplementary Figure 6a: Scatter plot of the ER+ signal/TNB signal  ratio from Affymetrix (50 ng total RNA input) experiment, vs ER+/TNB  ratio from NanoString experiment. Samples were from Case 2, and  prepared by Laboratory 1. | Supplementary Figure 6b: Scatter plot of the ER+ signal/TNB signal ratio  from Affymetrix (100 ng total RNA input) experiment, vs ER+/TNB ratio from  NanoString experiment. Samples were from Case 2, and prepared by  Laboratory 2. |
| c)  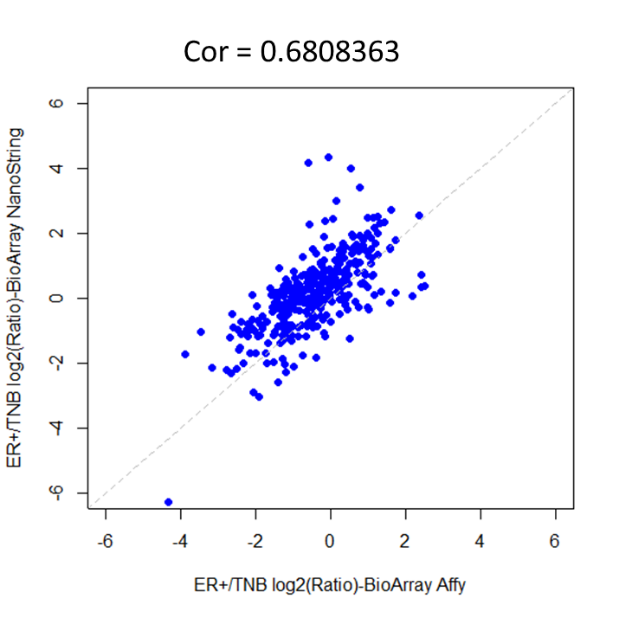 | d)  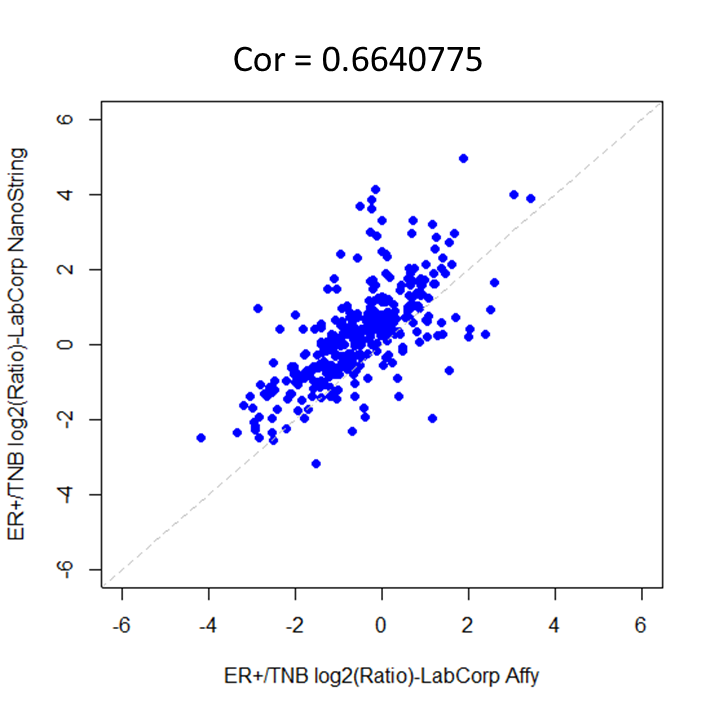 |
| Supplementary Figure 6c: Scatter plot of the ER+ signal/TNB signal  ratio from Affymetrix (50 ng total RNA input) experiment, vs ER+/TNB  ratio from NanoString experiment. Samples were from Case 4, and  prepared by Laboratory 1. | Supplementary Figure 6d: Scatter plot of the ER+ signal/TNB signal  ratio from Affymetrix (100 ng total RNA input) experiment, vs ER+/TNB  ratio from NanoString experiment. Samples were from Case 4, and  prepared by Laboratory 2. |

Supplementary Figure 6. Scatters plots comparing mRNA measurement using Affymetrix and NanoString platforms.
